# Pulsed Hyperoxia Acts on Plasmatic Advanced Glycation End Products and Advanced Oxidation Protein Products and Modulates Mitochondrial Biogenesis in Human Peripheral Blood Mononuclear Cells: A Pilot Study on the “Normobaric Oxygen Paradox”

**DOI:** 10.3390/ijms25042394

**Published:** 2024-02-18

**Authors:** Costantino Balestra, Sara Baldelli, Fabio Virgili, Michele Salvagno, Simona Mrakic-Sposta, Deborah Fratantonio

**Affiliations:** 1 Environmental, Occupational, Aging (Integrative) Physiology Laboratory, Haute Ecole Bruxelles-Brabant (HE2B), 1160 Brussels, Belgium; 2 Physical Activity Teaching Unit, Motor Sciences Department, Université Libre de Bruxelles (ULB), 1050 Brussels, Belgium; 3 DAN Europe Research Division (Roseto-Brussels), 1160 Brussels, Belgium; 4 Anatomical Research and Clinical Studies, Vrije Universiteit Brussels (VUB), 1090 Brussels, Belgium; 5 Department of Human Sciences and Promotion of the Quality of Life, IRCCS San Raffaele Pisana, San Raffaele Roma Open University, 00163 Rome, Italy; sara.baldelli@uniroma5.it; 6 Interuniversitary Consortium “National Institute for Bio-Structures and Bio-Systems”—I.N.B.B., 13, 00136 Rome, Italy; fvirgili@outlook.it; 7 Department of Intensive Care, Hôpital Universitaire de Bruxelles (HUB), 1070 Brussels, Belgium; michele.salvagno@ulb.be; 8 Institute of Clinical Physiology, National Research Council (CNR), 20162 Milan, Italy; simona.mrakicsposta@cnr.it; 9 Department of Medicine and Surgery, LUM University, S.S. 100 Km 18, 70100 Casamassima, Italy; fratantonio@lum.it

**Keywords:** human, peripheral blood mononuclear cells (PBMCs), reactive oxygen species (ROS), oxidative stress, Nrf2, PGC-1α, mitochondrial biogenesis, Prx3, targeted use of oxygen

## Abstract

The “normobaric oxygen paradox” (NOP) describes the response to the return to normoxia after a hyperoxic event, sensed by tissues as an oxygen shortage, up-regulating redox-sensitive transcription factors. We have previously characterized the time trend of oxygen-sensitive transcription factors in human PBMCs, in which the return to normoxia after 30% oxygen is sensed as a hypoxic trigger, characterized by hypoxia-induced factor (HIF-1) activation. On the contrary, 100% and 140% oxygen induce a shift toward an oxidative stress response, characterized by NRF2 and NF-kB activation in the first 24 h post exposure. Herein, we investigate whether this paradigm triggers Advanced Glycation End products (AGEs) and Advanced Oxidation Protein Products (AOPPs) as circulating biomarkers of oxidative stress. Secondly, we studied if mitochondrial biogenesis was involved to link the cellular response to oxidative stress in human PBMCs. Our results show that AGEs and AOPPs increase in a different manner according to oxygen dose. Mitochondrial levels of peroxiredoxin (PRX3) supported the cellular response to oxidative stress and increased at 24 h after mild hyperoxia, MH (30% O_2_), and high hyperoxia, HH (100% O_2_), while during very high hyperoxia, VHH (140% O_2_), the activation was significantly high only at 3 h after oxygen exposure. Mitochondrial biogenesis was activated through nuclear translocation of PGC-1α in all the experimental conditions. However, the consequent release of nuclear Mitochondrial Transcription Factor A (TFAM) was observed only after MH exposure. Conversely, HH and VHH are associated with a progressive loss of NOP response in the ability to induce TFAM expression despite a nuclear translocation of PGC-1α also occurring in these conditions. This study confirms that pulsed high oxygen treatment elicits specific cellular responses, according to its partial pressure and time of administration, and further emphasizes the importance of targeting the use of oxygen to activate specific effects on the whole organism.

## 1. Introduction

The appropriate availability and release of oxygen is fundamental to ensure cell functions, including cellular metabolism and growth. A relative decrease in O_2_ supply, or hypoxia, may induce acute and chronic pathologies such as cancer, cardiovascular disease, chronic obstructive pulmonary disease (COPD), metabolic disorders, and other stress responses [1,2]. However, some interesting positive benefits have been recently published using intermittent or “pulsed” hypoxia, showing interest in anti-aging, mitochondrial, or wellness and training effects [3,4,5,6,7,8,9,10]. Other research focuses on the hyperbaric side of hyperoxia, finding many benefits and positive outcomes in numerous diseases and other similar outcomes such as hypoxia in aging, post-traumatic stress disorders, or training, among others [11,12,13,14,15,16,17,18,19,20,21]. Conversely, chronic oxidative stress can be a consequence of excessive O_2_ exposure characterized by a persistent condition of an imbalance between the generation of ROS and the ability of the endogenous antioxidant system to detoxify them [22,23].

Oxidative stress induces glycoxidation reactions and modifications of free amino groups in proteins, resulting in the generation of Advanced Glycation End Products (AGEs) and Advanced Oxidation Protein Products (AOPPs). Both AGEs and AOPPs are considered circulating markers of oxidative stress in several pathologic conditions when an imbalance among oxidant and antioxidant responses occurs [24].

Although not intuitive, hypoxia also favors the formation of reactive oxygen species (ROS) and in the long run leads to an increase in oxidative stress, a condition that induces a strong prolonged inflammatory response [25,26,27]. In parallel, situations such as intense aerobic exercise require high amounts of O_2_ consumption, which consequently leads to an increased metabolism, an increase in ROS, and oxidative stress [28]. During light-intensity exercise, breathing oxygen-enriched mixtures, or, on the contrary, hypoxic air, produces similar reactions [29,30].

To date, it is known that cells respond to O_2_ fluctuations by activating two transcription factors: hypoxia-inducible factor-1α (HIF-1α) and Nrf2, which activate the transcription of multiple target genes [31]. Related to this, we demonstrated in previous works that pulsed hyperoxia induces a “hypoxic like” response, defined as a “normobaric oxygen paradox” (NOP) [32,33,34,35,36]. In particular, we observed that the return to a condition of normoxia after the induction of both mild and severe hyperoxia leads to an increase in HIF1 levels in human peripheral blood mononuclear cells (PBMCs) [33]. Furthermore, we also observed an increase in Nrf2 activation in the same experimental conditions [33]. Indeed, Nrf2 is activated in response to different types of stress related to a high flow of O_2_, inducing the repair or degradation of damaged macromolecules [37]. As mentioned before, Nrf2 is primarily known as an important key modulator of cellular metabolism and its target genes are involved in GSH synthesis, scavenging mitochondrial ROS (peroxiredoxin 3, Prx3), xenobiotic metabolism (NAD(P)H, quinone oxidoreductase 1, NQO1), and drug elimination (glutathione S-transferase, GST), thus maintaining ROS homeostasis [38,39].

More recently, new functions for Nrf2 have been highlighted concerning its ability to activate the transcription of genes involved in mitochondrial biogenesis. Mitochondria are double-membrane organelles that provide a dynamic and multifaceted role in cell signaling and metabolism. They also play an important role in cellular redox homeostasis through their involvement in ROS metabolism as one of the main sites of ROS production in the cell [40]. Mitochondria are organelles capable of dividing and growing in mass and size through the mechanism of mitochondrial biogenesis, which can be influenced by different factors, such as temperature, oxidative stress, and O_2_ fluctuations [41]. Mitochondrial biogenesis can be regulated by oxidative-stress-sensitive transcription factors such as NRF1, NRF2, and mitochondrial transcription factor A (TFAM) [23]. The synthesis of the latter is regulated by Nrf2. The mechanism is considered as follows: when Nrf2 binds to the EpRE sequences of NRF1, TFAM, together with the transcriptional coactivator peroxisome proliferator-activated receptor-c coactivator-1 α (PGC-1α), elicits the synthesis of new mitochondria [42].

Moreover, a regulatory mechanism between PGC-1α and Nrf2 has been shown, which is important to the enabling of physiological mitochondrial functions and oxidative metabolism in different tissues. In particular, upon metabolic stress (fasting) and/or oxidative imbalance (GSH depletion), p53 binds to the PPARGC1A sequence within the promoter of both human and mouse genes and positively regulates PGC-1α expression, which in turn co-activates Nrf2 gene expression and stimulates the up-regulation of antioxidant genes mitochondrial SOD2 and γGCS. Based on these premises, we hypothesize that PGC-1α and Nrf2 contribute together to counteract oxidative stress and induce mitochondrial biogenesis [43]. In fact, the protective roles of PGC-1α and Nrf2 in various pathologies have been highlighted [44]. To the best of our knowledge, in the context of molecular signaling induced by O_2_ fluctuations, there are no data available regarding the PGC-1α-Nrf2 pathway and TFAM.

Here, we report that NOP induces the activation of a redox-mediated PGC1-α-NRF2 pathway, intersects mitochondrial-protein activation (TFAM), and modulates mitochondrial cellular adaptive responses to redox imbalance.

## 2. Results

### 2.1. Pulsed Hyperoxia Increases AGEs and AOPP Plasma Levels in Humans

**Figure 1:**
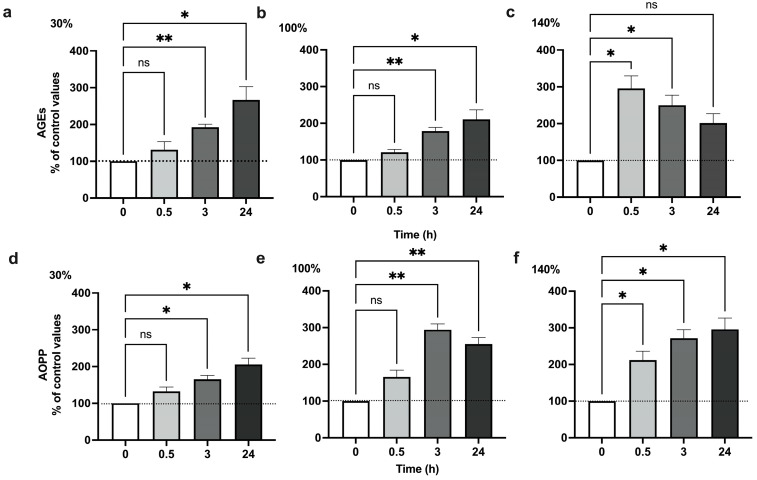
**AGE and AOPP production following 1 h hyperoxia.** Percentual changes in plasma levels of AGEs and AOPPs in healthy subjects exposed to mild hyperoxia (**a**,**d**), high hyperoxia (**b**,**e**), and very high hyperoxia (**c**,**f**), corresponding to 30%, 100%, and 140% O_2_, respectively, for 1 h. Measurements were taken at baseline (before O_2_ exposure), 30 min, 3 h, and 24 h after exposure by means of spectrofluorimetric and spectrophotometric detection. Data are reported in percentual changes from baseline (n = 4 subjects for each time point). Histograms’ colors are meant to ease reading and correspond to post exposure time. Measurements were performed in triplicate. * *p* < 0.05; ** *p* < 0.01; ns: non-significant; RM-ANOVA with Dunnet’s post hoc test.

We investigated whether O_2_ fluctuations induced oxidative stress markers. The plasma levels of AGEs and AOPPs were measured in healthy subjects after one hour of exposure to mild (MH), high (HH), and very high (VHH) hyperoxia, corresponding to 30%, 100%, and 140% O_2_, respectively. The exposure to 30% and 100% O_2_ significantly affected plasma levels of AGEs, with a clear increase at 3 h and 24 h after oxygen exposure (Figure 1a,b). Conversely, VHH exposure increased the plasma level of AGEs, peaking at 0.5 h after the return to normoxia (Figure 1c). The same trend was observed in AOPP plasma levels, with HH inducing a plasmatic increase at 3 and 24 h after the return to normoxia (Figure 1e), but also in the case of VHH, where a significant increase in AOPP occurs earlier (Figure 1f) but continues for a longer period, up to 24 h. These results confirm the induction of oxidative stress in human plasma during the NOP effect, with different responses in time.

### 2.2. Pulsed Hyperoxia Regulates Peroxiredoxin 3 Levels

**Figure 2:**
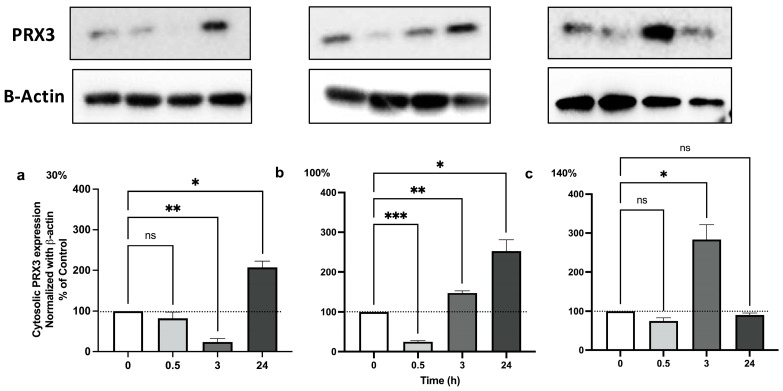
**Mitochondrial Prx3 protein expression following 1 h hyperoxia.** (**a**) Mild hyperoxia (30% O_2_); (**b**) high hyperoxia (100% O_2_); (**c**) very high hyperoxia (140% O_2_) before and after the recovery to normoxic conditions. In the above histograms, the picture shows a representative Western blot analysis. The density of immunoreactive bands was calculated using the software Quantity one (Bio-Rad, Hercules, CA, USA) and data are shown as a ratio of PRX3/B-actin. Results are expressed as percentual change (n = 4) (mean ± SEM) in comparison to baseline (0); Histograms’ colors are meant to ease reading and correspond to post exposure time. ns: not significant; *: *p* < 0.05, **: *p* < 0.01, ***: *p* < 0.001; RM-ANOVA with Dunnet’s post hoc test.

Nrf2 directly regulates mitochondrial ROS homeostasis by promoting detoxification of mitochondrial peroxides through Prx3 [39]. Hence, to determine the role of ROS in the induction of Nrf2-mediated Prx3 expression, we determined the effect of 30%, 100%, and 140% O_2_ administration on human PBMCs at the same time intervals indicated above. The Western blot analysis reported in Figure 2 shows that Prx3 was first diminished after 3 h and 30 min, respectively, for 30% and 100%, then showed a strong increase up to 24 h. The VHH exposure triggered a different reaction, showing a remarkable and unique peck 3 h post hyperbaric oxygen, reaching similar levels as mild oxygen exposures, but this did not last. These results suggest that the ROS-mediated activation of Nrf2 triggers the up-regulation of Prx3 protein expression, resulting in the protection of PBMCs from oxidative stress associated with the hyperoxic stimuli in a different way from normobaric oxygen levels, showing a prolonged, significant increase present after 24 h, while after 140% oxygen exposure, a single significant increase appears after 3 h.

### 2.3. Pulsed Hyperoxia Leads to PGC-1α Up-Regulation, but Only MH Results in TFAM Activation

**Figure 3:**
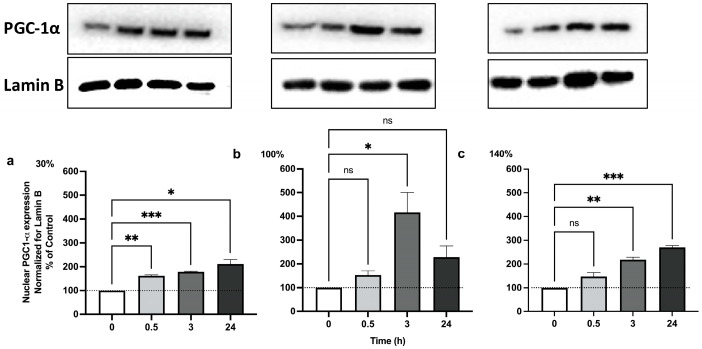
**PGC-1α nuclear translocation following 1 h hyperoxia:** (**a**) Mild hyperoxia (30% O_2_); (**b**) high hyperoxia (100% O_2_); (**c**) very high hyperoxia (140% O_2_) before and after the recovery to normoxic conditions. In the above histograms, the picture shows a representative Western blot analysis. The density of immunoreactive bands was calculated using the software Quantity One (Bio-Rad) and data are shown as ratio of PGC-1α/Lamin B. Results are expressed as percentual change (n = 4) (mean ± SEM) in comparison to baseline (0); Histograms’ colors are meant to ease reading and correspond to post exposure time. ns: not significant; *: *p* < 0.05, **: *p* < 0.01, ***: *p* < 0.001; RM-ANOVA with Dunnet’s post hoc test.

We analyzed the protein content of PGC-1α by Western blot analysis at 0.5, 3, and 24 h after 30%, 100%, and 140% O_2_ administration. As shown in Figure 3, PGC-1α significantly increased after 30 min, up to 24 h in MH (Figure 3a). HH treatment induced PGC-1α nuclear translocation only at 3 h (Figure 3b). VHH exposure was associated with an increase similar to what was observed after MH exposure (Figure 3c). These results indicate that the activation of PGC-1α precedes that of Nrf2, which is activated only at 3 h, suggesting a possible synergy between the two transcription factors. Similarly, HH treatment always causes an induction of PGC-1α at 30 min that goes along with the activation of Nrf2, which begins to increase at 30 min but peaks at 3 h. All together, these data suggest that Nrf2 is involved in the transcription and subsequent activation of PGC-1α in our experimental conditions.

**Figure 4:**
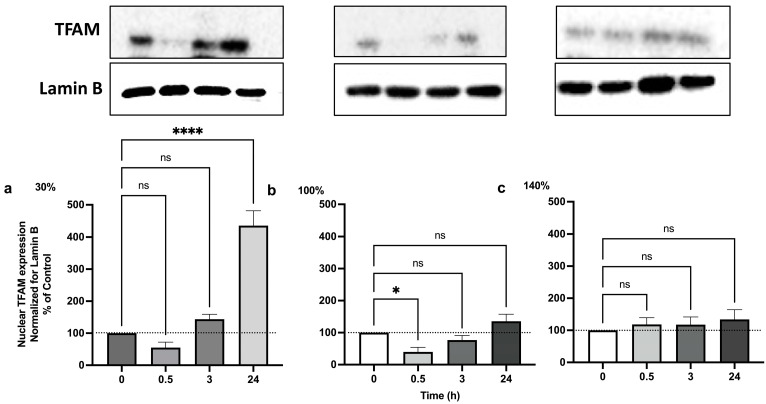
**TFAM activation following 1 h hyperoxia.** (**a**) Mild hyperoxia (30% O_2_); (**b**) high hyperoxia (100% O_2_); (**c**) very high hyperoxia (140% O_2_) before and after the recovery to normoxic conditions. In the above histograms, the picture shows a representative Western blot analysis. The density of immunoreactive bands was calculated using the software Quantity One (Bio-Rad) and data are shown as a ratio of TFAM/Lamin B. Results are expressed as percentual change (n = 4) (mean ± SEM) in comparison to baseline (0); Histograms’ colors are meant to ease reading and correspond to post exposure time. ns: not significant; *: *p* < 0.05, **** *p* < 0.0001; RM-ANOVA with Dunnet’s post hoc test.

Even though all tested oxygen exposures were associated with a significant activation of PGC-1α, only MH exposure resulted in a significant TFAM release and in the activation of mitochondrial biogenesis (Figure 4).

## 3. Materials and Methods

This study was conducted in accordance with the Declaration of Helsinki [45] and approved by the Academic Ethical Committee of Brussels (B200-2020-088). Every participant was fully informed of the procedures and was able to quit at any step of the procedure; written informed consent was obtained.

### 3.1. Experimental Protocol

After the obtention of full, written informed consent, twelve healthy non-smoking subjects (4 females and 8 males) enrolled for the experiment. These were physiotherapy students aged 21.8 ± 2.3 and 21.25 ± 2.1 years old (mean ± SD), with 1.75 m ± 6.6 height and 69.0 ± 8.7 kg weight. Participants were prospectively randomized into three groups, each comprising 4 persons, and exposed to different oxygen PO_2_ levels for 1 h. The first group received 30% O_2_ (0.3 bar; 300 hPa PO_2_) by means of an orofacial non-rebreather mask with a reservoir; the breathing gas flow (from a pressurized gas tank with the appropriate mixture) was set at 10 L/min, with care being taken to fit and tighten the mask on the subject’s face. Group two received 100% O_2_ (1.0 bar, 1000 hPa PO_2_) from an oxygen concentrator (NewLife Intensity, CAIRE Inc., Ball Ground, GA, USA) with a similar non-rebreathing mask setup. Group three received 140% O_2_ (1.400 bar, 1400 hPa PO_2_), using a one-person hyperbaric chamber (Biobarica, Buenos Aires, Argentina); the subject was breathing pure oxygen, 10 L/min, from a non-rebreathing mask inside the pressurized chamber.

Venous blood samples were collected at baseline (before oxygen exposure), 30 min, 3 h, and 24 h after exposure. Subjects were instructed not to take any medication or perform strenuous physical exercise 24 h before and, stay in altitude up to 2 weeks before and during the entire study protocol and until blood collection was complete.

Fifteen milliliters of blood were collected in ethylenediaminetetraacetic acid (EDTA). Human PBMCs were isolated from whole blood using a standard Histopaque-1077 (Sigma-Aldrich, Burlington, MA, USA) precipitation protocol, according to the manufacturer’s instruction, before oxygen breathing (time 0), as well as at 0.5, 3, and 24 h after exposure to hyperoxia. The absence, of hemolysis in plasma was confirmed by measuring the absorbance of plasma at 414 nm, using an absorbance of 0.2 as a cut-off.

### 3.2. Nuclear Lysate Preparation and Western Blotting Analysis

Nuclear lysate from human PBMCs was prepared as previously described by Fratantonio et al. [33]. In total, 20 μg of nuclear proteins, quantified with the Bradford method (Bio-Rad Laboratories Inc., Hercules, CA, USA), was separated by gel electrophoresis on 4–12% Bis-Tris Criterion XT precast gels (Bio-Rad Laboratories Inc., Hercules, CA, USA) and electroblotted onto polyvinylidene fluoride membranes (Amersham Pharmacia Biotech Inc., Piscataway, NJ, USA). Immunoblotting was performed with rabbit PGC-1α antibody (1:1000), rabbit Prx3 antibody (1:1000), and mouse anti-Lamin B antibody (1:1000) (Santa Cruz Biotechnology, Dallas, TX, USA), followed by peroxidase-conjugated secondary antibody HRP labeled goat anti-rabbit Ig (BD Pharmigen, San Diego, CA, USA) (1:5000) and goat anti-mouse IgM secondary antibody HRP conjugate (Thermo Scientific, Waltham, MA, USA) (1:10,000), and visualized with an Electrochemiluminescence (ECL) Western blotting system (Amersham Biosciences, Buckinghamshire, UK).

### 3.3. Plasma Analysis of AGEs and AOPPs

Determination of AGEs was based on the spectrofluorimetric detection as previously reported [46]. Briefly, blood plasma was diluted 1:50 with phosphate-buffered saline (PBS) pH 7.4 and fluorescence intensity was recorded at the emission maximum (~440 nm) upon excitation at 350 nm (spectrofluorometer, Shimadzu, Carlsbad, CA, USA). The serum concentration of AGEs was normalized to the total protein amount, determined by the Bradford assay and expressed in arbitrary units (AU) per gram of protein (AU/g prot).

Determination of AOPPs was based on spectrophotometric detection, as previously described [46]. Blood plasma (100 μL) or the same volume of chloramin T (0–100 μmol/L), for calibration, was diluted 1:5 with PBS pH 7.4. Subsequently, 25 μL of 1.16 M KI and 50 μL of acetic acid were added to the diluted solutions and absorbance was measured immediately at 340 nm (spectrofluorometer, Shimadzu, Carlsbad, CA, USA). The concentration of AOPPs is expressed in chloramine T units (μmol eq Cl T/L).

### 3.4. Statistical Analysis

All statistical tests were performed using a standard computer statistical package, GraphPad Prism version 9.00 for MacOS (GraphPad Software, San Diego, CA, USA).

Normality of data was verified by means of Kolmogorov–Smirnoff or Shapiro–Wilk tests, allowing us to assume a Gaussian distribution. Since each participant was their own control, data were analyzed using repeated measures ANOVA with Dunnett’s multiple comparison or Tukey’s post hoc test, and if the Gaussian distribution was not ascertained, Friedman with Dunn’s post-test was preferred.

A threshold of *p* < 0.05 was considered statistically significant. All data are presented as mean ± standard error on the mean (SEM).

## 4. Discussion

In this study, we identified changes in the plasma levels of AGEs and AOPPs in healthy human subjects after different O_2_ concentration exposures. We also examined the involvement of the mitochondrial response to oxygen-fluctuation-induced oxidative stress by the activation of PRX3 and the modulation of mitochondrial biogenesis.

Previous observations indicate that breathing 30% and 100% oxygen elicited a significant increase in plasmatic ROS, with a peak at 8 h after oxygen breathing, while the exposure to 140% (1.4 ATA) oxygen was associated with an increase in plasmatic ROS at 2 h after the return to normoxia [33,47,48].

In line with these results, we observed a consequent increase in oxidation end products in human plasma. In particular, the exposure to 30% and 100% oxygen increased the plasma levels of AGEs (Figure 1a,b) at 3 and 24 h, while 140% oxygen induced a higher significant increase in AGEs faster, with a peak at 0.5 h, and progressively returned close to baseline levels after 24 h (Figure 1c).

A similar trend was also observed for AOPPs for 30% and 100% oxygen (Figure 1c,d), while for 140% oxygen a progressive significant increase starting from 0.5 h up to 24 h (Figure 1e) showed an opposite trend to AGEs for the same exposure.

Advanced glycation end products (AGEs) are derived from nonenzymatic glycation occurring between the reactive carbonyl group of a reducing sugar and nucleic acids, lipids, or proteins, causing underlying tissue damage [49]. In addition to nonenzymatic glycation, AGEs can also be formed through the polyol pathway and lipid peroxidation. AGEs produce reactive oxygen (ROS) and nitrogen (RNS) species, as well as oxidative stress and inflammation [50]. We already acknowledged such oxidative stress increases with several markers, but without measuring the Maillard reaction; these results confirm our previous findings of higher oxidative stress for hyperbaric oxygen exposures at 1.4 ATA (140%) and 2.5 ATA after a single session [47] but, in the present results, with a faster recovery for 140%. These results were expected since we already found lipidic peroxidation after all levels of oxygen exposure, from hypoxia to hyperbaric hyperoxia, sometimes still present 48 h post exposure [27,47,51].

Interestingly, Advanced Oxidation Protein Products (AOPPs), a biomarker of oxidant-mediated protein damage which can increase ROS levels, follow the same tendency of AGEs except for the 140% (1.4 ATA) exposure. We are unable to fully explain this trend; however, we observed a diminished cellular response compared to other exposures at 1.4 ATA [52]. It is known that oxygen exposure elicits “Oxy-Inflammation”, a term proposed by Valacchi et al. [53] for a condition characterized by the alteration of systemic inflammation and severely compromised redox balance, and we can understand why several hyperbaric sessions (at 2.5 ATA) are needed to adapt and counteract inflammatory or oxidative stress [54]. Lower oxygen levels or even “hyperbaric air” have faster cellular hormetic responses [33,55].

The increase in oxidative stress and its metabolic consequences was confirmed by measuring, in human PBMCs, the cytosolic level of Peroxiredoxin 3 (Prx3), a mitochondrial antioxidant protein which serves as a major antioxidant enzyme and eliminates approximately 90% of H_2_O_2_ in mitochondria [56].

We previously demonstrated that the administration of pulsed hyperoxia induces a “paradoxical” hypoxic response characterized by Nrf2 activation [33]. In particular, we observed an increase in Nrf2 nuclear protein levels at 3 h when 30% and 140% O_2_ were administered. Nrf2 levels remained constant up to 24 h. Conversely, the administration of 100% O_2_ was associated with an increase in Nrf2 levels, starting at 30 min after the return to normoxia and levelling back to the baseline at 24 h [33].

The understanding of the roles of Nrf2 and PGC-1α in the regulation of oxidative stress and in maintaining mitochondrial homeostasis could provide novel information to support the treatment of various pathologies associated with O_2_ fluctuations or to develop a novel approach to oxygen use [52,57].

One of the accepted mechanisms is the following: PGC-1α activates Nrf2 via the inhibition of GSK3b. GSK3b is inactivated by p38, which is positively regulated by PGC-1α. Therefore, the PGC-1α/p38/GSK3b/Nrf2 cascade is the most probable pathway for mitochondrial DNA transcription [58].

**Figure 5:**
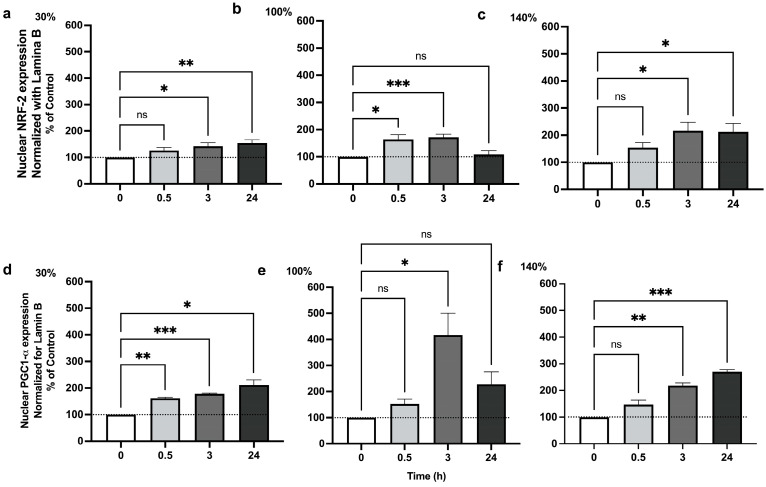
**NRF2 and PGC-1α nuclear translocation 1 h hyperoxia.** Percentual changes in plasma level of NRF2 (redrawn from Fratantonio et al. 2021 [33]) and PGC-1α in healthy subjects exposed to mild hyperoxia (**a**,**d**), high hyperoxia (**b**,**e**), and very high hyperoxia (**c**,**f**), corresponding to 30%, 100%, and 140% O_2_, respectively, for 1 h. Measurements were taken at baseline (before O_2_ exposure), 30 min, 3 h, and 24 h after exposure by means of spectrofluorimetric and spectrophotometric detection. Data are reported in percentual changes from baseline (n = 4 subjects for each time point). Histograms’ colors are meant to ease reading and correspond to post exposure time. Measurements were performed in triplicate. * *p* < 0.05; ** *p* < 0.01; *** *p* < 0.001; ns: non-significant; RM-ANOVA with Dunnet’s post hoc test.

It is also possible that Nrf2 and PGC-1α form a feedback loop together [38]; our results show a largely parallel evolution of both (see Figure 5a–c).

In our experimental conditions, despite the activation of PGC-1α, we observed a significant activation of mitochondrial biogenesis, in terms of TFAM expression, only following the exposure to mild hyperoxia. This suggests that the ability of PGC-1α to activate NFR2 and TFAM occurs when oxidative stress levels can be handled by antioxidant cellular responses. When oxidative stress levels overcome the cellular capacity to counteract oxidative stress, as previously reported, NF-kB activation takes over [33] and the mitochondrial biogenesis response is lost.

Moreover, erythropoietin (EPO) was found to activate mitochondrial biogenesis [59]. Therefore, EPO can potentially activate both the Nrf2 and PGC-1α cascades. We have shown that in the NOP mechanism, varying oxygen levels below hyperbaric doses can, after one single session, activate EPO production up to 36 h. On the contrary, a single session of hyperbaric oxygen showed a reduction in plasmatic erythropoietin for 24 h [32,34,60,61].

## 5. Conclusions

This study emphasizes the importance of targeting the use of oxygen to activate specific cellular responses [52,57]. Further analysis is needed to understand how several sessions of different levels of oxygen breathing, for different durations, and with different in-between recovery periods, will modulate such responses.

The Renaissance physician Paracelsus noted that, “Nothing is without poison—the poison is in the dose”. The contemporary interpretation of this statement is that dose and effect move together in a predictably linear fashion and lower exposures to a hazardous compound will generally generate lower risks.

Our results show that this “linearity” on reduced risk is not only present on the toxicity side, but also on the elicited response. In fact, it seems that in the first 24 h following a session, lower oxygen concentrations act more positively than higher levels of hyperoxia on mitochondrial biogenesis factors.

We are aware that the number of subjects is small, and this limits the “power” of our study. Nonetheless, we consider this report a pilot study. Moreover, our data allow a better characterization of the complex spectrum of cell responses to pulsed oxygen concentration at the whole-organism level, resulting in a proof of principle study indicating the involvement of mitochondrial activity in the managing of oxidative stress. Additional studies are surely warranted to corroborate and confirm our observation.

**Figure 6:**
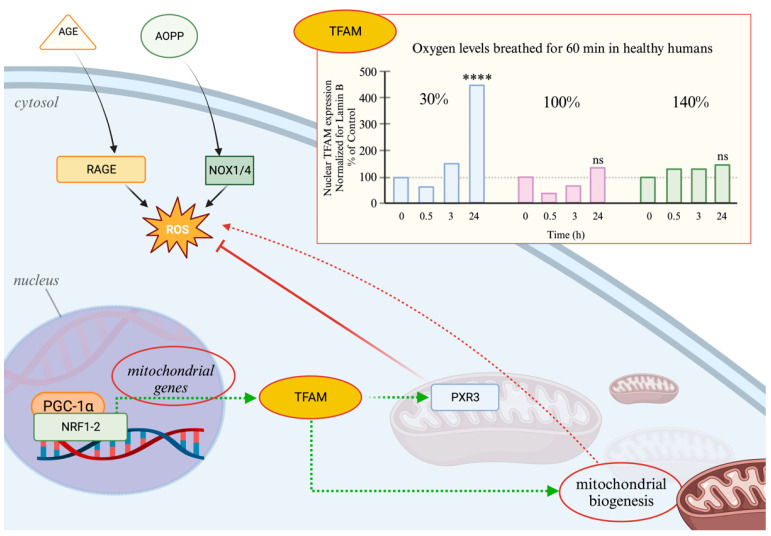
The bar graph in the upper right corner demonstrates the effect of NOP on redox-mediated PGC1α-NRF1-2 signaling, assessed by the upregulation of TFAM. It displays the relative expression levels of TFAM following 60 min of exposure to 30%, 100%, and 140% oxygen concentrations at various intervals (baseline, 0.5, 3, 24 h); **** *p* < 0.0001; ns: non-significant.

When an important production of ROS is present within the cytosol, (left part of the figure), pathways activated by Advanced Glycation End-products (AGEs) and Advanced Oxidation Protein Products (AOPPs) are initiated. AGEs trigger the AGE receptor (RAGE), leading to further formation of ROS and proinflammatory cytokines. AOPPs may induce oxidative stress through NADPH oxidases (Nox). As depicted in the lower left corner, NRF1-2 interaction, along with the transcriptional coactivator PGC-1α, triggers the synthesis of TFAM, facilitating mitochondrial biogenesis (indicated by the green dotted lines). This process indirectly contributes to ROS production since mitochondria serve as a significant ROS source. At the same time when a level of ROS is not to high and intermittent, TFAM maintains mitochondrial ROS balance by increasing the production of Prx3 (green dotted lines) thus facilitating the detoxification of mitochondrial peroxides via Prx3 (shown by the red dotted line from Prx3 to ROS) (Figure 6).

## Data Availability

The datasets used and analyzed during the current study are available from the corresponding author on reasonable request.

## Abbreviations

NOP	normobaric oxygen paradox
ATA	Atmosphere absolute
PBMCs	peripheral blood mononuclear cells
NF-kB	Nuclear factor kappa-light-chain-enhancer of activated B
FiO_2_	Inspired Fraction of Oxygen
GSH	Glutathione
H_2_O_2_	Hydrogen peroxide
HBOT	Hyperbaric oxygen therapy
AGEs	Advanced Glycation End Products
AOPPs	Advanced Oxidation Protein Products
NOx	Nitric oxide metabolites
NRF2	Nuclear Factor Erythroid 2 Related—Factor 2
ROS	reactive oxygen species
PRX3	peroxiredoxin
TFAM	Nuclear mitochondrial transcription factor A
PGC-1α	proliferator-activated receptor-c coactivator-1 α
GSH	Glutathione
EpRE	Electrophile Response Element
RAGE	Receptor for Advanced Glycation End-products

## References

[1] Chen P.S., Chiu W.T., Hsu P.L., Lin S.C., Peng I.C., Wang C.Y., Tsai S.J. (2020). Pathophysiological implications of hypoxia in human diseases. J. Biomed. Sci..

[2] Luo Z., Tian M., Yang G., Tan Q., Chen Y., Li G., Zhang Q., Li Y., Wan P., Wu J. (2022). Hypoxia signaling in human health and diseases: Implications and prospects for therapeutics. Signal Transduct. Target. Ther..

[3] Raberin A., Burtscher J., Citherlet T., Manferdelli G., Krumm B., Bourdillon N., Antero J., Rasica L., Malatesta D., Brocherie F. (2023). Women at Altitude: Sex-Related Physiological Responses to Exercise in Hypoxia. Sports Med..

[4] Panza G.S., Burtscher J., Zhao F. (2023). Intermittent hypoxia: A call for harmonization in terminology. J. Appl. Physiol..

[5] Ehrenreich H., Gassmann M., Poustka L., Burtscher M., Hammermann P., Sirén A.L., Nave K.A., Miskowiak K. (2023). Exploiting moderate hypoxia to benefit patients with brain disease: Molecular mechanisms and translational research in progress. Neuroprotection.

[6] Burtscher M., Burtscher J. (2023). MFN2: Shaping mitochondria and cardiac adaptations to hypoxia. Acta Physiol..

[7] Burtscher J., Raberin A., Brocherie F., Malatesta D., Manferdelli G., Citherlet T., Krumm B., Bourdillon N., Antero J., Rasica L. (2023). Recommendations for Women in Mountain Sports and Hypoxia Training/Conditioning. Sports Med..

[8] Burtscher J., Pasha Q., Chanana N., Millet G.P., Burtscher M., Strasser B. (2023). Immune consequences of exercise in hypoxia: A narrative review. J. Sport Health Sci..

[9] Burtscher J., Hohenauer E., Burtscher M., Millet G.P., Egg M. (2023). Environmental and behavioral regulation of HIF-mitochondria crosstalk. Free Radic. Biol. Med..

[10] Burtscher J., Citherlet T., Camacho-Cardenosa A., Camacho-Cardenosa M., Raberin A., Krumm B., Hohenauer E., Egg M., Lichtblau M., Müller J. (2023). Mechanisms underlying the health benefits of intermittent hypoxia conditioning. J. Physiol..

[11] Cannellotto M., Yasells García A., Landa M.S. (2024). Hyperoxia: Effective Mechanism of Hyperbaric Treatment at Mild-Pressure. Int. J. Mol. Sci..

[12] Ablin J.N., Lang E., Catalogna M., Aloush V., Hadanny A., Doenyas-Barak K., Finci S., Polak N., Fishlev G., Korin C. (2023). Hyperbaric oxygen therapy compared to pharmacological intervention in fibromyalgia patients following traumatic brain injury: A randomized, controlled trial. PLoS ONE.

[13] Hadanny A., Hachmo Y., Rozali D., Catalogna M., Yaakobi E., Sova M., Gattegno H., Abu Hamed R., Lang E., Polak N. (2022). Effects of Hyperbaric Oxygen Therapy on Mitochondrial Respiration and Physical Performance in Middle-Aged Athletes: A Blinded, Randomized Controlled Trial. Sports Med. Open.

[14] Hadanny A., Efrati S. (2022). Editorial: Hyperbaric oxygen and the brain. Front. Neurol..

[15] Doenyas-Barak K., Catalogna M., Kutz I., Levi G., Hadanny A., Tal S., Daphna-Tekoha S., Sasson E., Shechter Y., Efrati S. (2022). Hyperbaric oxygen therapy improves symptoms, brain's microstructure and functionality in veterans with treatment resistant post-traumatic stress disorder: A prospective, randomized, controlled trial. PLoS ONE.

[16] Catalogna M., Sasson E., Hadanny A., Parag Y., Zilberman-Itskovich S., Efrati S. (2022). Effects of hyperbaric oxygen therapy on functional and structural connectivity in post-COVID-19 condition patients: A randomized, sham-controlled trial. Neuroimage Clin..

[17] Cannellotto M., Duarte M., Keller G., Larrea R., Cunto E., Chediack V., Mansur M., Brito D.M., García E., Di Salvo H.E. (2022). Hyperbaric oxygen as an adjuvant treatment for patients with COVID-19 severe hypoxaemia: A randomised controlled trial. Emerg. Med. J..

[18] Shapira R., Gdalyahu A., Gottfried I., Sasson E., Hadanny A., Efrati S., Blinder P., Ashery U. (2021). Hyperbaric oxygen therapy alleviates vascular dysfunction and amyloid burden in an Alzheimer’s disease mouse model and in elderly patients. Aging.

[19] Hadanny A., Forer R., Volodarsky D., Daniel-Kotovsky M., Catalogna M., Zemel Y., Bechor Y., Efrati S. (2021). Hyperbaric oxygen therapy induces transcriptome changes in elderly: A prospective trial. Aging.

[20] Hachmo Y., Hadanny A., Mendelovic S., Hillman P., Shapira E., Landau G., Gattegno H., Zrachya A., Daniel-Kotovsky M., Catalogna M. (2021). The effect of hyperbaric oxygen therapy on the pathophysiology of skin aging: A prospective clinical trial. Aging.

[21] Hachmo Y., Hadanny A., Abu Hamed R., Daniel-Kotovsky M., Catalogna M., Fishlev G., Lang E., Polak N., Doenyas K., Friedman M. (2020). Hyperbaric oxygen therapy increases telomere length and decreases immunosenescence in isolated blood cells: A prospective trial. Aging.

[22] Ottolenghi S., Rubino F.M., Sabbatini G., Coppola S., Veronese A., Chiumello D., Paroni R. (2019). Oxidative Stress Markers to Investigate the Effects of Hyperoxia in Anesthesia. Int. J. Mol. Sci..

[23] Lee H.C., Yin P.H., Chi C.W., Wei Y.H. (2002). Increase in mitochondrial mass in human fibroblasts under oxidative stress and during replicative cell senescence. J. Biomed. Sci..

[24] Marrocco I., Altieri F., Peluso I. (2017). Measurement and Clinical Significance of Biomarkers of Oxidative Stress in Humans. Oxid. Med. Cell Longev..

[25] McGarry T., Biniecka M., Veale D.J., Fearon U. (2018). Hypoxia, oxidative stress and inflammation. Free Radic. Biol. Med..

[26] Mrakic-Sposta S., Gussoni M., Marzorati M., Porcelli S., Bosco G., Balestra C., Montorsi M., Lafortuna C., Vezzoli A. (2023). The “ON-OFF” Switching Response of Reactive Oxygen Species in Acute Normobaric Hypoxia: Preliminary Outcome. Int. J. Mol. Sci..

[27] Leveque C., Mrakic Sposta S., Theunissen S., Germonpré P., Lambrechts K., Vezzoli A., Gussoni M., Levenez M., Lafère P., Guerrero F. (2023). Oxidative Stress Response Kinetics after 60 Minutes at Different Levels (10% or 15%) of Normobaric Hypoxia Exposure. Int. J. Mol. Sci..

[28] He F., Li J., Liu Z., Chuang C.C., Yang W., Zuo L. (2016). Redox Mechanism of Reactive Oxygen Species in Exercise. Front. Physiol..

[29] Bosco G., Paganini M., Giacon T.A., Oppio A., Vezzoli A., Dellanoce C., Moro T., Paoli A., Zanotti F., Zavan B. (2021). Oxidative Stress and Inflammation, MicroRNA, and Hemoglobin Variations after Administration of Oxygen at Different Pressures and Concentrations: A Randomized Trial. Int. J. Environ. Res. Public Health.

[30] Balestra C., Lambrechts K., Mrakic-Sposta S., Vezzoli A., Levenez M., Germonpre P., Virgili F., Bosco G., Lafere P. (2021). Hypoxic and Hyperoxic Breathing as a Complement to Low-Intensity Physical Exercise Programs: A Proof-of-Principle Study. Int. J. Mol. Sci..

[31] D'Aiuto N., Hochmann J., Millan M., Di Paolo A., Bologna-Molina R., Sotelo Silveira J., Arocena M. (2022). Hypoxia, acidification and oxidative stress in cells cultured at large distances from an oxygen source. Sci. Rep..

[32] Salvagno M., Coppalini G., Taccone F.S., Strapazzon G., Mrakic-Sposta S., Rocco M., Khalife M., Balestra C. (2022). The Normobaric Oxygen Paradox-Hyperoxic Hypoxic Paradox: A Novel Expedient Strategy in Hematopoiesis Clinical Issues. Int. J. Mol. Sci..

[33] Fratantonio D., Virgili F., Zucchi A., Lambrechts K., Latronico T., Lafere P., Germonpre P., Balestra C. (2021). Increasing Oxygen Partial Pressures Induce a Distinct Transcriptional Response in Human PBMC: A Pilot Study on the “Normobaric Oxygen Paradox”. Int. J. Mol. Sci..

[34] Rocco M., D'Itri L., De Bels D., Corazza F., Balestra C. (2014). The “normobaric oxygen paradox”: A new tool for the anesthetist?. Minerva Anestesiol..

[35] De Bels D., Theunissen S., Devriendt J., Germonpre P., Lafere P., Valsamis J., Snoeck T., Meeus P., Balestra C. (2012). The ‘normobaric oxygen paradox’: Does it increase haemoglobin?. Diving Hyperb. Med..

[36] Cimino F., Balestra C., Germonpre P., De Bels D., Tillmans F., Saija A., Speciale A., Virgili F. (2012). Pulsed high oxygen induces a hypoxic-like response in human umbilical endothelial cells and in humans. J. Appl. Physiol..

[37] Fratantonio D., Cimino F., Speciale A., Virgili F. (2018). Need (more than) two to Tango: Multiple tools to adapt to changes in oxygen availability. Biofactors.

[38] Baldelli S., Aquilano K., Ciriolo M.R. (2013). Punctum on two different transcription factors regulated by PGC-1α: Nuclear factor erythroid-derived 2-like 2 and nuclear respiratory factor 2. Biochim. Biophys. Acta.

[39] Ma Q. (2013). Role of nrf2 in oxidative stress and toxicity. Annu. Rev. Pharmacol. Toxicol..

[40] Rigoulet M., Yoboue E.D., Devin A. (2011). Mitochondrial ROS generation and its regulation: Mechanisms involved in H_2_O_2_ signaling. Antioxid. Redox Signal..

[41] Jornayvaz F.R., Shulman G.I. (2010). Regulation of mitochondrial biogenesis. Essays Biochem..

[42] Tufekci K.U., Civi Bayin E., Genc S., Genc K. (2011). The Nrf2/ARE Pathway: A Promising Target to Counteract Mitochondrial Dysfunction in Parkinson’s Disease. Parkinsons Dis..

[43] Aquilano K., Baldelli S., Pagliei B., Cannata S.M., Rotilio G., Ciriolo M.R. (2013). p53 orchestrates the PGC-1alpha-mediated antioxidant response upon mild redox and metabolic imbalance. Antioxid. Redox Signal..

[44] Villavicencio Tejo F., Quintanilla R.A. (2021). Contribution of the Nrf2 Pathway on Oxidative Damage and Mitochondrial Failure in Parkinson and Alzheimer’s Disease. Antioxidants.

[45] World Medical A. (2013). World Medical Association Declaration of Helsinki: Ethical principles for medical research involving human subjects. JAMA.

[46] Ruggeri R.M., Vicchio T.M., Cristani M., Certo R., Caccamo D., Alibrandi A., Giovinazzo S., Saija A., Campenni A., Trimarchi F. (2016). Oxidative Stress and Advanced Glycation End Products in Hashimoto’s Thyroiditis. Thyroid.

[47] Leveque C., Mrakic Sposta S., Theunissen S., Germonpré P., Lambrechts K., Vezzoli A., Bosco G., Lévénez M., Lafère P., Guerrero F. (2023). Oxidative Stress Response Kinetics after 60 Minutes at Different (1.4 ATA and 2.5 ATA) Hyperbaric Hyperoxia Exposures. Int. J. Mol. Sci..

[48] Leveque C., Mrakic-Sposta S., Lafere P., Vezzoli A., Germonpre P., Beer A., Mievis S., Virgili F., Lambrechts K., Theunissen S. (2022). Oxidative Stress Response’s Kinetics after 60 Minutes at Different (30% or 100%) Normobaric Hyperoxia Exposures. Int. J. Mol. Sci..

[49] Schmitt A., Schmitt J., Münch G., Gasic-Milencovic J. (2005). Characterization of advanced glycation end products for biochemical studies: Side chain modifications and fluorescence characteristics. Anal. Biochem..

[50] Henning C., Glomb M.A. (2016). Pathways of the Maillard reaction under physiological conditions. Glycoconj. J..

[51] Levenez M., Lambrechts K., Mrakic-Sposta S., Vezzoli A., Germonpre P., Pique H., Virgili F., Bosco G., Lafere P., Balestra C. (2022). Full-Face Mask Use during SCUBA Diving Counters Related Oxidative Stress and Endothelial Dysfunction. Int. J. Environ. Res. Public Health.

[52] Balestra C., Arya A.K., Leveque C., Virgili F., Germonpre P., Lambrechts K., Lafere P., Thom S.R. (2022). Varying Oxygen Partial Pressure Elicits Blood-Borne Microparticles Expressing Different Cell-Specific Proteins-Toward a Targeted Use of Oxygen?. Int. J. Mol. Sci..

[53] Valacchi G., Virgili F., Cervellati C., Pecorelli A. (2018). OxInflammation: From Subclinical Condition to Pathological Biomarker. Front. Physiol..

[54] de Wolde S.D., Hulskes R.H., de Jonge S.W., Hollmann M.W., van Hulst R.A., Weenink R.P., Kox M. (2022). The Effect of Hyperbaric Oxygen Therapy on Markers of Oxidative Stress and the Immune Response in Healthy Volunteers. Front. Physiol..

[55] MacLaughlin K.J., Barton G.P., Braun R.K., MacLaughlin J.E., Lamers J.J., Marcou M.D., Eldridge M.W. (2023). Hyperbaric air mobilizes stem cells in humans; a new perspective on the hormetic dose curve. Front. Neurol..

[56] Cox A.G., Winterbourn C.C., Hampton M.B. (2009). Mitochondrial peroxiredoxin involvement in antioxidant defence and redox signalling. Biochem. J..

[57] Balestra C., Kot J. (2021). Oxygen: A Stimulus, Not “Only” a Drug. Medicina.

[58] Choi H.I., Kim H.J., Park J.S., Kim I.J., Bae E.H., Ma S.K., Kim S.W. (2017). PGC-1α attenuates hydrogen peroxide-induced apoptotic cell death by upregulating Nrf-2 via GSK3β inactivation mediated by activated p38 in HK-2 Cells. Sci. Rep..

[59] Larsen S., Dam Søndergård S., Eg Sahl R., Frandsen J., Morville T., Dela F., Helge J.W. (2021). Acute erythropoietin injection increases muscle mitochondrial respiratory capacity in young men: A double-blinded randomized crossover trial. J. Appl. Physiol..

[60] Balestra C., Germonpre P. (2011). Increasing EPO using the normobaric oxygen paradox: A ‘not so simple’ task. Acta Physiol..

[61] Balestra C., Germonpre P., Poortmans J.R., Marroni A. (2006). Serum erythropoietin levels in healthy humans after a short period of normobaric and hyperbaric oxygen breathing: The “normobaric oxygen paradox”. J. Appl. Physiol..

